# Characteristics and Factors Associated with Death among Patients Hospitalized for Severe Fever with Thrombocytopenia Syndrome, South Korea, 2013

**DOI:** 10.3201/eid2110.141928

**Published:** 2015-10

**Authors:** Jaeseung Shin, Donghyok Kwon, Seung-Ki Youn, Ji-Hyuk Park

**Affiliations:** Korea Centers for Disease Control and Prevention, Cheongju-si, South Korea (J. Shin, D. Kwon, S.-K. Youn, J.-H. Park);; Dongguk University College of Medicine, Gyeongju-si, South Korea (J.-H. Park)

**Keywords:** Severe fever with thrombocytopenia syndrome, viruses, phlebovirus, RNA virus, *Bunyaviridae*, bunyavirus, tickborne infections, vector-borne infections, *Haemaphysalis* *longicornis*, ribavirin, intravenous immunoglobulin, plasmapheresis, continuous renal replacement therapy, fungal pneumonia, cerebral hemorrhage, South Korea

## Abstract

In South Korea, nationwide surveillance for severe fever with thrombocytopenia syndrome (SFTS) began during 2013. Among 301 surveillance cases, 35 hospitalized case-patients in 25 areas were confirmed by using virologic testing, and 16 (46%) case-patients subsequently died. The SFTS cases occurred during May–November and peaked during June (9 cases, 26%). The incidence of SFTS was higher in the southern regions of South Korea. Age and neurologic symptoms, including decreased level of consciousness and slurred speech, were heavily associated with death; neurologic symptoms during the first week after disease onset were also associated with death. Although melena was common among patients who died, no other hemorrhagic manifestations were substantively more common among those who died. No effective treatments, including ribavirin, were identified. Expansion of SFTS surveillance to include the outpatient sector and development of an antibody test would enhance completeness of SFTS detection in South Korea.

## Abstract 2

Surveillance for this emerging disease should be expanded to the outpatient setting.

Severe fever with thrombocytopenia syndrome (SFTS) is an emerging infectious disease that is caused by a novel phlebovirus in the *Bunyaviridae* family. This virus has been named severe fever with thrombocytopenia syndrome virus (SFTSV). The disease was first reported during 2009 in China, where it is most prevalent in the Hunan, Hubei, and Shandong provinces, which are located at a similar latitude to that of South Korea and Japan (*1*). SFTS is mainly transmitted to humans by SFTSV-infected ticks, most frequently *Haemaphysalis longicornis* (*2*). However, person-to-person transmission by direct contact with infected blood or body fluid has also been reported (*3*,*4*).

The clinical symptoms of SFTS include fever; gastrointestinal (GI) symptoms (e.g., diarrhea and vomiting); leukocytopenia and thrombocytopenia; bleeding tendency; and neurologic symptoms. The incubation period for SFTS is 1–2 weeks, and illness progresses through 3 stages: fever, multiorgan dysfunction, and convalescence. No effective treatment for SFTS has been established (*5*).

In South Korea, the laboratory diagnostic system for SFTS was established during March 2013, and the presence of SFTSV among *H. longicornis* ticks was confirmed by using samples from ticks that were collected during 2011–2012 (*6*). Based on the nationwide surveillance reports for SFTS and its designation as a notifiable infectious disease, the first SFTS case was retrospectively confirmed in May 2013, after the patient’s death in 2012 (*7*). However, because of the novel nature of this system, the clinical and demographic characteristics of patients in South Korea infected with SFTS are not well understood. Therefore, this study evaluated the characteristics and factors that were associated with SFTS-related fatalities in South Korea, as reported during 2013.

## Methods

### Surveillance System and Case Definition

A passive hospital-based surveillance system for SFTS was initiated nationwide in South Korea during March 2013. Physicians were advised to request SFTSV testing for patients with known clinical manifestations of SFTS, including fever (body temperature >38.0°C), thrombocytopenia, leukocytopenia, and GI symptoms. Each patient’s enrollment for SFTSV testing was dependent on the physician’s clinical suspicion of SFTS. Serum samples from 301 patients admitted on the basis of physician referrals at 125 hospitals throughout Korea were collected and tested. Case-patients were defined as patients who had clinical symptoms and were confirmed to have SFTSV by virologic testing at the Korea Centers for Disease Control and Prevention (KCDC). This study did not require an institutional ethics review because it was conducted under the Infectious Disease Control and Prevention Act in South Korea.

### Laboratory Testing

All acute-phase serum samples were tested to detect the SFTSV medium segment gene by one-step reverse transcription PCR, by using DiaStar 2× OneStep reverse transcription PCR Pre-Mix Kit (SolGent, Daejeon, South Korea), as described (*8*). The PCR primers were MF3 (5′-GATGAGATGGTCCATGCTGATTCT-3′) and MR2 (5′-CTCATGGGGTGGAATGTCCTCAC-3′). The PCR conditions were an initial step of 30 min at 50°C for reverse transcription; 15 min at 95°C for denaturation; 35 cycles of 20 s at 95°C, 40 s at 58°C, and 30 s at 72°C; and a final extension step of 5 min at 72°C.

### Epidemiologic Investigation

We also performed an epidemiologic investigation of patients in whom SFTS was suspected immediately after the SFTSV testing was requested by their physicians. KCDC Epidemic Intelligence Service officers interviewed the patients and their physicians using a standardized questionnaire that evaluated demographic characteristics, exposure history, clinical symptoms, and laboratory results. Patients were also questioned regarding their exposure history within the month before their onset of symptoms. Multiple activities were documented, including agricultural and forestry work, mountain climbing, and visits to a family grave. The date of the tick bite was self-reported when the patient was aware of a bite or if the patient participated in only 1 exposure-related activity over a short period of time. In addition, after the death or discharge of confirmed case-patients during September 2013–January 2014, the clinical course and prognosis were investigated by reviewing medical records. The date for clinical suspicion of SFTS was recorded as the date reported to the KCDC.

### Statistical Analysis

We obtained median regional population data during 2013 from Statistics Korea (http://www.kostat.go.kr) to calculate the regional incidences during 2013. These incidences were then overlaid on a map of South Korea by using bio-geographic information system software (DIVA-GIS 7.5; http://www.diva-gis.org). We used the Fisher exact test or the Mann-Whitney U test to compare the prognoses of patients with SFTS. All statistical analyses were performed by using SAS software version 9.2 (SAS Institute, Cary, NC, USA), and statistical significance was set at p<0.05.

### Results

During 2013, a total of 36 hospitalized patients were confirmed to have SFTS. One case was excluded because the patient’s disease onset occurred during 2012. Thus, 35 cases from 20 hospitals were included in our analysis.

### Demographic Characteristics

**Figure 1:**
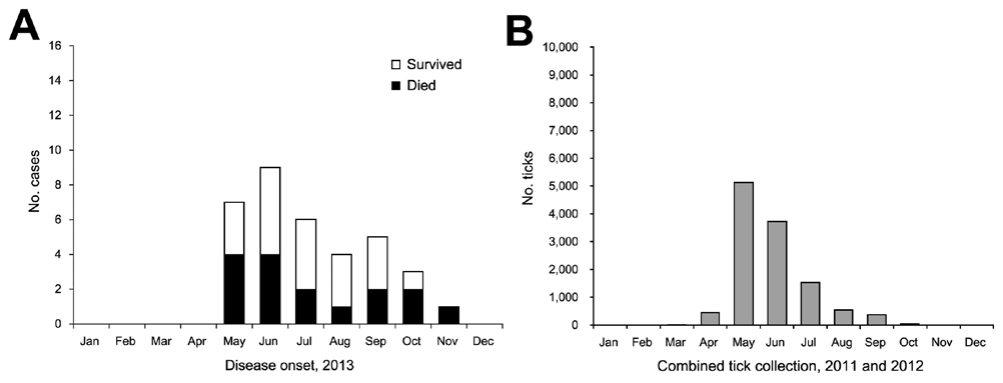
Comparison of epidemic curve for severe fever with thrombocytopenia syndrome cases identified during 2013 and the number of *Haemaphysalis longicornis* ticks collected per month during 2011 and 2012, South Korea. A) Number of cases of severe fever with thrombocytopenia syndrome, by month of onset. B) Combined number of *H. longicornis* ticks collected, by month (*6*).

Symptom onset occurred during May–November 2013; a peak of symptoms among 9 (26%) patients occurred in June. Similar patterns were observed among the SFTS case-patients in our study (Figure 1, panel A) and the number of *H. longicornis* ticks that were collected each month by Park et al. during 2011–2012 (*6*) (Figure 1, panel B). Peaks of symptoms among patients occurred in June 2013, and peaks of the number of ticks collected occurred in May 2011 and May 2012. 

**Figure 2:**
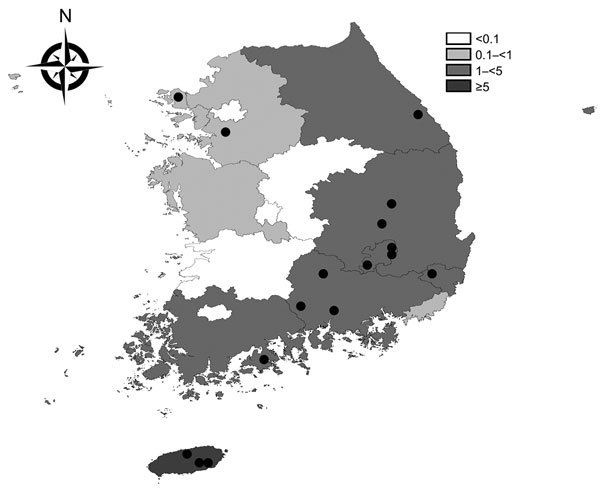
Geographic distribution of severe fever with thrombocytopenia syndrome cases, South Korea, 2013. Shading indicates incidence of cases per 1 million residents. Black circles indicate the approximate residential regions of the 16 case-patients who died.

The overall incidence of SFTS during 2013 was 0.7 cases/1 million persons. Geographic differences were documented in the incidences in 25 cities; higher incidences were observed in the southern regions, including Jeju Province (Figure 2).

The case-patient group comprised 18 women (51%) and 17 men (49%). The median age was 69 (range 28–84) years, and age was significantly higher among those who died (range 62–82 years; p = 0.026). Twenty-six (74%) case-patients resided in rural areas, and 25 (71%) were farmers. A trace of a tick bite was found on 11 (31%) case-patients, and 4 (13%) had recognized the tick bite before their hospital admission. The most common exposure-related activity was agricultural work (n = 20, 61%), followed by forestry work (n = 7, 21%), mountain climbing (n = 6, 18%), and visiting a family member’s grave (n = 3, 9%). The agricultural work was performed in dry fields by 16 (80%) of 20 case-patients and forestry work was done in orchards by 5 (71%) of 7 case-patients (Table 1).

**Table 1:** Characteristics of hospitalized case-patients with confirmed severe fever with thrombocytopenia syndrome, by outcome, South Korea, 2013

Characteristics	No. (% or range)			p value
	Total, n = 35	Died, n = 16	Recovered, n = 19	
Sex				
M	17 (49)	7 (44)	10 (53)	0.738
F	18 (51)	9 (56)	9 (47)	
Age, y	69 (28–84)	73.5 (62–82)	61 (28–84)	0.026
Residence				
Rural	26 (74)	11 (69)	15 (79)	0.700
Urban	9 (26)	5 (31)	4 (21)	
Occupation				
Farmers	25 (71)	12 (75)	13 (68)	0.723
Others	10 (29)	4 (25)	6 (32)	
Medical history				
Diabetes mellitus	11 (31)	5 (31)	6 (32)	1.000
Hypertension	18 (51)	9 (60)	9 (47)	0.738
Hepatitis B	2 (6)	2 (13)	0 (0)	0.202
Tuberculosis	1 (3)	1 (6)	0 (0)	0.457
Trace of tick bite	11 (31)	5 (31)	6 (32)	1.000
Recognition of tick bite, n = 32	4 (13)	2 (14)	2 (11)	1.000
Exposure-related activities, n = 33†				
Agricultural work	20 (61)	9 (60)	11 (61)	1.000
Forestry work	7 (21)	4 (27)	3 (17)	0.674
Mountain climbing	6 (18)	1 (7)	5 (28)	0.186
Visits to family members' grave	3 (9)	2 (13)	1 (6)	0.579
Time elapsed, d				
From onset to admission	4 (1–9)	3.5 (1–7)	4 (2–9)	0.659‡
From onset to clinical suspicion§	7 (3–22)	6.5 (3–11)	7 (3–22)	0.612‡
From onset to death	ND¶	10.5 (4–32)	ND	ND
From onset to discharge	ND	ND	18 (10–49)	ND
*ND, no data available. †Fisher exact test was used to compare groups, unless otherwise indicated. ‡Multiple responses were allowed. §Mann-Whitney U test was used to compare groups. ¶Days to clinical suspicion were determined based on the day of report to the Korea Centers for Disease Control and Prevention.				

The incubation period was available for 8 patients; the median was 9 (range 5–16) days. Median time from symptom onset to hospital admission was 4 (range 1–9) days and from symptom onset to clinical suspicion was 7 (range 3–22) days. We found no substantive differences in time from symptom onset to admission and to clinical suspicion between the group of persons who died and those who recovered. Twenty-four (69%) of the 35 patients with SFTS were admitted to intensive care units, and 16 (46%) died. The median survival time among the 16 was 10.5 (range 4–32) days; 11 (69%) patients died within 2 weeks after symptom onset. Among patients who survived, median time from symptom onset to discharge was 18 (range 10–49) days (Table 1).

The cohort included 1 family cluster, which consisted of an uncle and nephew. They did not live in the same village and did not have contact with each other after symptom onset. However, within 2 weeks before symptom onset, both had visited the same family member’s grave on a mountain and had mowed the grass.

### Clinical Characteristics and Prognoses

The clinical characteristics of the 35 SFTS case-patients during the course of the illness are shown in Table 2. All patients experienced fever or chills, and other common symptoms included GI symptoms (n = 33, 94%), neurologic symptoms (n = 27, 77%), fatigue (n = 26, 74%), myalgia (n = 19, 54%), and hemorrhagic manifestations (n = 12, 34%). Among the 32 patients with medical records for the first 7 days after disease onset, all patients had a fever within 7 days of symptom onset. A high fever (≥39°C) was documented for 7 (22%) patients, although no significant difference was observed related to occurrence of fever among those who died and those who recovered (p = 0.195). Lymphadenopathy was identified by physical examination on admission on the neck, face, left thigh, left axilla, or left inguinal area of 5 (14%) patients.

**Table 2:** Clinical characteristics of hospitalized case-patients with confirmed severe fever with thrombocytopenia syndrome, by outcome, South Korea, 2013*

Characteristics	No. (%) case-patients			p value†
	Total, n = 35	Died, n = 16	Recovered, n = 19	
Fever/chills	35 (100)	16 (100)	19 (100)	1.000
Fever, n = 32				
≤39.0°C	25 (78)	9 (64)	16 (89)	0.195
>39.0°C	7 (22)	5 (36)	2 (11)	NA
Fatigue	26 (74)	11 (69)	15 (79)	0.700
Myalgia	19 (54)	9 (56)	10 (53)	1.000
Headache	12 (34)	4 (25)	8 (42)	0.476
Cough	7 (20)	3 (19)	4 (21)	1.000
Sputum	9 (26)	3 (19)	6 (32)	0.460
Lymphadenopathy on physical examination	5 (14)	1 (6)	4 (21)	0.347
Lymph node enlargement found by CT, n = 32	20 (63)	7 (54)	13 (68)	0.473
Gastrointestinal symptoms				
Overall	33 (94)	15 (94)	18 (95)	1.000
Anorexia	21 (60)	8 (50)	13 (68)	0.317
Nausea	16 (46)	5 (31)	11 (58)	0.176
Abdominal pain	9 (26)	6 (38)	3 (16)	0.245
Diarrhea	22 (63)	12 (75)	10 (53)	0.293
Vomiting	15 (43)	6 (38)	9 (47)	0.734
Diarrhea/vomiting	26 (74)	13 (81)	13 (68)	0.460
Neurologic symptoms				
Overall	27 (77)	15 (94)	12 (63)	0.047
Within 7 d after disease onset	20 (57)	14 (88)	6 (32)	0.002
Slurred speech	9 (26)	7 (44)	2 (11)	0.050
Decreased level of consciousness	26 (74)	15 (94)	11 (58)	0.022
Convulsions	8 (23)	6 (38)	2 (11)	0.105
Hemorrhagic manifestations				
Overall	12 (34)	8 (50)	4 (21)	0.090
Gross hematuria	2 (6)	1 (6)	1 (5)	1.000
Petechiae	3 (9)	1 (6)	2 (11)	1.000
Gingival bleeding	5 (14)	3 (19)	2 (11)	0.642
Hematemesis	2 (6)	1 (6)	1 (5)	1.000
Hematochezia	1 (3)	1 (6)	0 (0)	0.457
Melena	4 (11)	4 (25)	0 (0)	0.035
Treatment				
Ribavirin	9 (26)	6 (38)	3 (16)	0.245
IVIG	7 (20)	5 (31)	2 (11)	0.208
Plasmapheresis	7 (20)	4 (25)	3 (16)	0.677
CRRT	10 (29)	9 (56)	1 (5)	0.002

*NA, not applicable; CT, computed tomography; IVIG, intravenous immunoglobulin; CRRT, continuous renal replacement therapy. †Fisher exact test was used to compare groups.

The most common GI symptoms among all patients were diarrhea (n = 22, 63%), anorexia (n = 21, 60%), nausea (n = 16, 46%), and vomiting (n = 15, 43%). Both diarrhea and vomiting were reported for 26 (74%) patients; most (25/26, 96%) experienced diarrhea or vomiting within 1 week after symptom onset, and 18 (51%) patients had diarrhea or vomiting at the time of admission. However, no substantive differences in the occurrence of GI symptoms were observed for those who died compared with those who recovered.

Decreased level of consciousness (n = 26, 74%) was the most frequent neurologic symptom, followed by slurred speech (n = 9, 26%) and convulsion (n = 8, 23%). Neurologic symptoms occurred at a median of 6 days after symptom onset (range 2–10 days). On admission, 3 patients (9%) had neurologic symptoms. The group of case-patients who died had a significantly higher number of members who had neurologic symptoms (p = 0.047) and exhibited significantly more frequent neurologic symptoms that occurred within 7 days (p = 0.002). Decreased level of consciousness (p = 0.050) and slurred speech (p = 0.022) were significantly more common among those who died, although the frequency of convulsion was similar for both groups (p = 0.105).

The only hemorrhagic manifestation that occurred with a significant difference among those who died and those who recovered was melena (p = 0.035). None of the treatments of SFTS appeared to be effective, including the use of ribavirin, intravenous immunoglobulin, plasmapheresis, or continuous renal replacement therapy. Fungal pneumonia (n = 2, 6%) and cerebral hemorrhage (n = 2, 6%) were observed as complications among those who died.

### Laboratory Features and Prognoses

The laboratory data from the 32 patients with available medical records for the first 7 days after symptom onset are shown in Table 3. All patients had thrombocytopenia (platelets <150 × 10^9^/L) and leukocytopenia (leukocytes <4 × 10^9^ cells/L) on admission. The median minimum platelet count was marginally lower among those who died (34 × 10^9^/L), compared with that among those who recovered (47.5 × 10^9^/L; p = 0.054). The maximum serum aspartate aminotransferase, lactate dehydrogenase (LDH), creatinine kinase, and creatinine kinase myocardial b fraction levels were similar for both groups. However, the maximum alkaline phosphatase (ALP) levels during the first week after symptom onset were significantly higher for patients who died than for those who recovered (213 U/L vs. 79 U/L; p=0.017).

**Table 3:** Laboratory features of hospitalized case-patients with confirmed severe fever with thrombocytopenia syndrome during the first week after onset, by outcome, South Korea, 2013*

Laboratory tests	Median (range)			p value†
	Total, n = 32	Died, n = 14	Recovered, n = 18	
Platelet count, × 10^9^/L‡	38 (15–113)	34 (15–113)	47.5 (29–107)	0.054
Leukocyte count, × 10^9^ cells/L‡	1.5 (0.7–3.0)	1.5 (0.7–3.0)	1.5 (0.7–2.7)	0.925
ANC, × 10^6^ cells/L, n = 30‡	969 (125–3,292)	1,042 (380–2,367)	920 (125–3,292)	0.637
Hemoglobin, g/L‡	12.9 (9.2–16.4)	12.1 (9.2–15.1)	13.5 (9.8–16.4)	0.193
aPTT, s, n = 30	54 (35–97)	60 (35–97)	54 (36–73)	0.400
AST, U/L, n = 31	242 (63–4,567)	420 (103–4,567)	223 (63–2,145)	0.109
ALT, U/L, n = 31	77 (27–1,432)	156 (28–1,432)	63 (27–477)	0.186
Total bilirubin, mg/dL, n = 31	0.5 (0.2–4.0)	0.6 (0.3–4.0)	0.5 (0.2–1.3)	0.109
Amylase, U/L, n = 26	84 (40–333)	78 (49–163)	90 (40–333)	0.540
Lipase, U/L, n = 20	98 (38–692)	92 (38–369)	117 (40–692)	0.370
CK, U/L, n = 21	570 (67–4,362)	428 (158–4,362)	676 (67–2,760)	0.651
CK-MB, U/L, n = 18	6.9 (0.3–300.0)	4.8 (1.4–300.0)	7.6 (0.3–35.0)	0.762
LDH, U/L, n = 28	908 (279–4,564)	1,799 (279–4,564)	893 (348–3,920)	0.260
ALP, U/L, n = 31	119 (44–1,586)	213 (53–1,586)	79 (44–510)	0.017

*ANC, absolute neutrophil count; aPTT, activated partial thromboplastin time; AST, aspartate aminotransferase; ALT, alanine aminotransferase; CK, creatinine kinase; CK-MB, creatinine kinase myocardial b fraction; LDH, lactate dehydrogenase; ALP, alkaline phosphatase. †Mann-Whitney U test was used to compare groups. ‡Minimum value.

## Discussion

We found that SFTS cases occurred throughout South Korea, although the incidence was higher in the southern part of the country. Among the various clinical manifestations, neurologic symptoms (overall and within 7 days after disease onset) were substantially more frequent among the case-patients who died, although GI symptoms and hemorrhagic manifestations (except melena) were more frequent among those who recovered. No effective treatment, including ribavirin, was identified.

The higher incidence of SFTS in the southern part of South Korea was particularly notable for Jeju Province, which is the largest island and the most southern part of South Korea. The prevalence of *H. longicornis* ticks in Jeju Province is among of the highest in South Korea (*9*,*10*); high temperatures are conducive to the survival and breeding of this species (*11*), and Jeju Province has the highest average temperature in South Korea, related to its low latitude (*12*). Furthermore, a study of ticks that were collected during 2011–2012 in South Korea reported that the minimum infection rates for SFTSV in *H. longicornis* ticks were higher in the southern part of the country (*6*).

Although the SFTS case-fatality rate in South Korea (46%) was higher than those that have been reported by using SFTS data from China (6%–30%) (*1*,*13*), it was similar to the rate (55%) that was reported in a retrospective tracing study in Japan (*14*). However, after the introduction of the China surveillance system in 2009, a decreasing trend in the case-fatality rate in that country has been observed (*13*), which may be supported by the increased capability to detect mild SFTS cases. Age was associated with a prognosis of death in our study, and other studies of hospitalized patients in China with SFTS have reported similar findings (*15*–*17*). However, in our study, the median ages for those who died versus those who survived (73.5 years and 61 years, respectively) tended to be higher than those among the patients in China (62.1–74 years and 52.9–60 years, respectively) (*15*–*17*). This older age might partially explain why we observed a higher case-fatality rate compared with previous reports. However, we did not find any person-to-person clusters of infection, and found only 1 family cluster, although these patterns have been reported in China (*3*,*18*).

The median time from symptom onset to admission was 4 days and from onset to clinical suspicion was 7 days. This finding indicates that there is a delay in diagnosis and appropriate care, which is likely related to lack of clinical experience with SFTS among physicians in South Korea. Nevertheless, despite the lack of a statistically significant relationship between this time period and death, a delayed diagnosis of SFTS could affect the prognosis of the patients.

The fever stage of SFTS occurs during the first week after disease onset and is characterized by the sudden onset of fever and GI symptoms (*19*); our findings were similar. However, we found that only 22% of those patients had a high fever (≥39°C), compared with 73% of hospitalized SFTS patients in China (*19*). Diarrhea was observed in 63% of patients in our study, which is higher than the 27%–57% rates that were observed in previous studies of hospitalized patients with SFTS (*1*,*19*,*20*). However, 43% of the patients in our study experienced vomiting, which is similar to the 31%–47% rates that were reported in the previous studies (*1*,*19*,*20*).

We found that neurologic symptoms were associated with a greater likelihood of death, and similar findings have been reported in previous studies of hospitalized patients in China (*19*,*20*). Moreover, we observed a rapid progression and increased frequency of neurologic symptoms during the fever stage among the patients who died, suggesting that early neurologic symptoms portend a fatal outcome.

Hemorrhagic manifestations have been linked to fatal outcomes among SFTS patients in China (*19*,*20*). However, we did not observe a similar association, with the exception of melena. Differences between the health care systems in South Korea and China, especially regarding the use of appropriate platelet transfusions, may partially explain the differences regarding hemorrhagic manifestations.

The lowest platelet counts during the first week were not substantively different between those who recovered and those who died. However, one study has reported that low platelet counts on admission or during the entire course were associated with SFTS severity (*20*). Therefore, it is possible that our relatively small number of cases may obscure the association of the lowest platelet counts with death among patients in South Korea.

According to 2 studies in China, elevated LDH levels on admission or during the entire course of illness were significantly associated with a fatal outcome (*15*,*20*). However, we did not observe a significant association between the highest LDH levels during the first week or at the time of admission and death (data not shown). We did observe significantly higher ALP levels during the first week among those who died, although a study of ALP levels on admission reported similar levels between those who died and those who recovered on the basis of multivariate analysis findings (*15*). Additional studies are needed to evaluate the relationships between a prognosis of death and high levels of LDH or ALP.

Our study has several limitations. First, mild cases of SFTS might have been missed, given that all of our case-patients were hospitalized. Thus, the true case-fatality rate in South Korea is likely lower than our reported value, related to case ascertainment bias. Second, because of the limited number of SFTS cases during 2013, we could not perform multivariate analysis for the prognosis. Therefore, our findings should be interpreted with caution. Third, because of the case series design, we could not analyze the risk factors for SFTS infection.

In conclusion, the clinical symptoms of SFTS in South Korea appear similar to those experienced by hospitalized SFTS patients in China. Older age and early neurologic symptoms were associated with a fatal outcome in studies in both countries. Expansion of SFTS surveillance into the outpatient sector, along with the development and incorporation of an SFTSV antibody test into the case detection algorithm, would detect milder cases and enhance completeness of SFTS case detection in South Korea.

## References

[1] Yu XJ, Liang MF, Zhang SY, Liu Y, Li JD, Sun YL, Fever with thrombocytopenia associated with a novel bunyavirus in China. N Engl J Med. 2011;364:1523–32. 10.1056/NEJMoa1010095 21410387 PMC3113718

[2] Park SW, Han MG, Yun SM, Park C, Lee WJ, Ryou J. Severe fever with thrombocytopenia syndrome virus, South Korea, 2013. Emerg Infect Dis. 2014;20:1880–2. 10.3201/eid2011.140888 25341085 PMC4214315

[3] Bao CJ, Guo XL, Qi X, Hu JL, Zhou MH, Varma JK, A family cluster of infections by a newly recognized bunyavirus in eastern China, 2007: further evidence of person-to-person transmission. Clin Infect Dis. 2011;53:1208–14. 10.1093/cid/cir732 22028437

[4] Gai Z, Liang M, Zhang Y, Zhang S, Jin C, Wang SW, Person-to-person transmission of severe fever with thrombocytopenia syndrome bunyavirus through blood contact. Clin Infect Dis. 2012;54:249–52. 10.1093/cid/cir776 22095565 PMC3245727

[5] Liu S, Chai C, Wang C, Am S, Lv H, He H, Systematic review of severe fever with thrombocytopenia syndrome: virology, epidemiology, and clinical characteristics. Rev Med Virol. 2014;24:90–102. 10.1002/rmv.1776 24310908 PMC4237196

[6] Park SW, Song BG, Shin EH, Yun SM, Han MG, Park MY, Prevalence of severe fever with thrombocytopenia syndrome virus in *Haemaphysalis longicornis* ticks in South Korea. Ticks Tick Borne Dis. 2014;5:975–7. 10.1016/j.ttbdis.2014.07.020 25164614

[7] Kim KH, Yi J, Kim G, Choi SJ, Jun KI, Kim NH, Severe fever with thrombocytopenia syndrome, South Korea, 2012. Emerg Infect Dis. 2013;19:1892–4. 10.3201/eid1911.130792 24206586 PMC3837670

[8] Yun SM, Lee WG, Ryou J, Yang SC, Park SW, Roh JY, Severe fever with thrombocytopenia syndrome virus in ticks collected from humans, South Korea, 2013. Emerg Infect Dis. 2014;20:1358–61. 10.3201/eid2008.131857 25061851 PMC4111194

[9] Ko S, Kang JG, Kim SY, Kim HC, Klein TA, Chong ST, Prevalence of tick-borne encephalitis virus in ticks from southern Korea. J Vet Sci. 2010;11:197–203. 10.4142/jvs.2010.11.3.197 20706026 PMC2924480

[10] Yun SM, Song BG, Choi W, Park WI, Kim SY, Roh JY, Prevalence of tick-borne encephalitis virus in ixodid ticks collected from the Republic of Korea during 2011–2012. Osong Public Health Res Perspect. 2012;3:213–21. 10.1016/j.phrp.2012.10.004 PMC3747658 24159517

[11] Yano Y, Shiraishi S, Uchida TA. Effects of temperature on development and growth in the tick, *Haemaphysalis longicornis.* Exp Appl Acarol. 1987;3:73–8. 10.1007/BF01200415 3453334

[12] Chung YS, Yoon MB. Interpretation of recent temperature and precipitation trends observed in Korea. Theor Appl Climatol. 2000;67:171–80. 10.1007/s007040070006

[13] Ding F, Zhang W, Wang L, Hu W, Soares Magalhaes RJ, Sun H, Epidemiologic features of severe fever with thrombocytopenia syndrome in China, 2011–2012. Clin Infect Dis. 2013;56:1682–3. 10.1093/cid/cit100 23429379

[14] Takahashi T, Maeda K, Suzuki T, Ishido A, Shigeoka T, Tominaga T, The first identification and retrospective study of severe fever with thrombocytopenia syndrome in Japan. J Infect Dis. 2014;209:816–27. 10.1093/infdis/jit603 24231186 PMC7107388

[15] Liu W, Lu QB, Cui N, Li H, Wang LY, Liu K, Case-fatality ratio and effectiveness of ribavirin therapy among hospitalized patients in China who had severe fever with thrombocytopenia syndrome. Clin Infect Dis. 2013;57:1292–9. 10.1093/cid/cit530 23965284

[16] Sun Y, Jin C, Zhan F, Wang X, Liang M, Zhang Q, Host cytokine storm is associated with disease severity of severe fever with thrombocytopenia syndrome. J Infect Dis. 2012;206:1085–94. 10.1093/infdis/jis452 22904342

[17] Zhang YZ, He YW, Dai YA, Xiong Y, Zheng H, Zhou DJ, Hemorrhagic fever caused by a novel Bunyavirus in China: pathogenesis and correlates of fatal outcome. Clin Infect Dis. 2012;54:527–33. 10.1093/cid/cir804 22144540

[18] Tang X, Wu W, Wang H, Du Y, Liu L, Kang K, Human-to-human transmission of severe fever with thrombocytopenia syndrome bunyavirus through contact with infectious blood. J Infect Dis. 2013;207:736–9. 10.1093/infdis/jis748 23225899

[19] Gai ZT, Zhang Y, Liang MF, Jin C, Zhang S, Zhu CB, Clinical progress and risk factors for death in severe fever with thrombocytopenia syndrome patients. J Infect Dis. 2012;206:1095–102. 10.1093/infdis/jis472 22850122

[20] Deng B, Zhou B, Zhang S, Zhu Y, Han L, Geng Y, Clinical features and factors associated with severity and fatality among patients with severe fever with thrombocytopenia syndrome bunyavirus infection in northeast China. PLoS ONE. 2013;8:e80802 . 10.1371/journal.pone.0080802 24236203 PMC3827460

